# *N*-[2-(4-Chloro­phen­yl)-5-methyl-4-oxo-1,3-thia­zolidin-3-yl]pyridine-3-carboxamide

**DOI:** 10.1107/S1600536811007136

**Published:** 2011-03-02

**Authors:** Mehmet Akkurt, Ísmail Çelik, Hale Demir, Sumru Özkırımlı, Orhan Büyükgüngör

**Affiliations:** a Department of Physics, Faculty of Sciences, Erciyes University, 38039 Kayseri, Turkey; b Department of Physics, Faculty of Arts and Sciences, Cumhuriyet University, 58140 Sivas, Turkey; c Department of Pharmaceutical Chemistry, Faculty of Pharmacy, Istanbul University, 34116 Beyazit, Istanbul, Turkey; d Department of Physics, Faculty of Arts and Sciences, Ondokuz Mayıs University, 55139 Samsun, Turkey

## Abstract

The title compound, C_16_H_14_ClN_3_O_2_S, crystallizes with two mol­ecules in the asymmetric unit. In the 1,3-thia­zolidine rings, the carbonyl O atoms, the S atoms, the methyl groups and the ring carbon attached to the methyl groups are disordered with occupancy ratios of 0.509 (7):0.491 (7) in one mol­ecule and 0.464 (14):0.536 (14) in the other. The crystal structure is stabilized by inter­molecular N—H⋯N, C—H⋯O hydrogen bonds and C—H⋯Cl inter­actions. In addition, there is a π–π stacking inter­action [centroid–centroid distance = 3.794 (3) Å] between the benzene and pyridine rings.

## Related literature

For the biological and pharmaceutical properties of nicotinamide derivatives, see: Barreca *et al.* (2003 ▶); Chen *et al.* (2009 ▶); Güzeldemirci *et al.* (2010 ▶); Gaudineau & Auclair (2004 ▶); Jaju *et al.* (2009 ▶); Joy *et al.* (2005 ▶); Karali *et al.* (1998 ▶); Mitchell *et al.* (2009 ▶); Ozkirimli *et al.* (2009 ▶); Patel & Shaikh (2010 ▶); Vigorita *et al.* (2003 ▶).
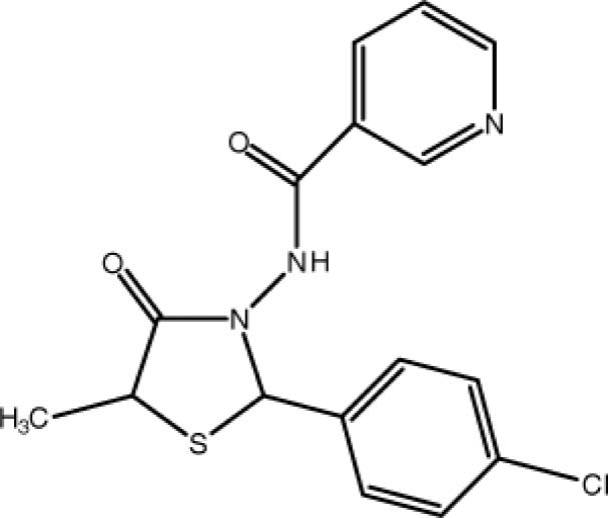


## Experimental

### 

#### Crystal data

C_16_H_14_ClN_3_O_2_S
*M* *_r_* = 347.82
Triclinic, 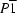

*a* = 11.4481 (7) Å
*b* = 12.0276 (7) Å
*c* = 12.5556 (7) Å
α = 91.798 (5)°
β = 100.282 (5)°
γ = 98.068 (5)°
*V* = 1681.39 (17) Å^3^
*Z* = 4
Mo *K*α radiation
μ = 0.36 mm^−1^
*T* = 296 K
0.75 × 0.46 × 0.24 mm

#### Data collection

Stoe IPDS 2 diffractometer
Absorption correction: integration (*X-RED32*; Stoe & Cie, 2002 ▶) *T*_min_ = 0.772, *T*_max_ = 0.918
27641 measured reflections
7719 independent reflections
3832 reflections with *I* > 2σ(*I*)
*R*_int_ = 0.079

#### Refinement

*R*[*F* ^2^ > 2σ(*F* ^2^)] = 0.079
*wR*(*F* ^2^) = 0.225
*S* = 1.04
7719 reflections
472 parameters
22 restraints
H atoms treated by a mixture of independent and constrained refinement
Δρ_max_ = 0.66 e Å^−3^
Δρ_min_ = −0.36 e Å^−3^

### 

Data collection: *X-AREA* (Stoe & Cie, 2002 ▶); cell refinement: *X-AREA*; data reduction: *X-RED32* (Stoe & Cie, 2002 ▶); program(s) used to solve structure: *SHELXS97* (Sheldrick, 2008 ▶); program(s) used to refine structure: *SHELXL97* (Sheldrick, 2008 ▶); molecular graphics: *ORTEP-3* (Farrugia, 1997 ▶); software used to prepare material for publication: *WinGX* (Farrugia, 1999 ▶).

## Supplementary Material

Crystal structure: contains datablocks global, I. DOI: 10.1107/S1600536811007136/qm2002sup1.cif

Structure factors: contains datablocks I. DOI: 10.1107/S1600536811007136/qm2002Isup2.hkl

Additional supplementary materials:  crystallographic information; 3D view; checkCIF report

## Figures and Tables

**Table 1:** Hydrogen-bond geometry (Å, °)

*D*—H⋯*A*	*D*—H	H⋯*A*	*D*⋯*A*	*D*—H⋯*A*
N2—H*N*2⋯N6^i^	0.85 (3)	2.09 (3)	2.932 (5)	168 (3)
N5—H*N*5⋯N3^ii^	0.86 (4)	2.16 (4)	2.950 (5)	153 (4)
C5—H5⋯O2^iii^	0.93	2.42	3.245 (6)	147
C14—H14⋯O1*A*^ii^	0.93	2.58	3.160 (14)	121
C16—H16⋯O3*B*^ii^	0.93	2.50	3.242 (15)	136
C17—H17⋯O4^iv^	0.93	2.50	3.367 (6)	155
C26*B*—H26*D*⋯Cl1^v^	0.96	2.76	3.373 (13)	123
C31—H31⋯O3*B*^vi^	0.93	2.45	3.139 (13)	131

Symmetry codes: (i) 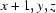; (ii) 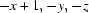; (iii) 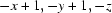; (iv) 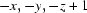; (v) 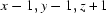; (vi) 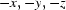.

**Fig. 1.**
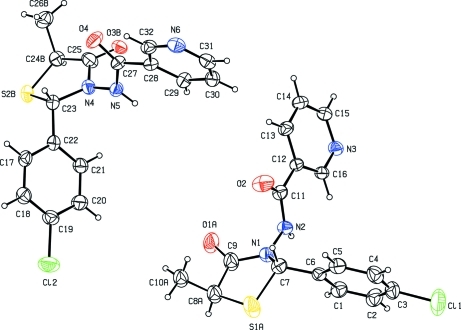
View of the major components of the two disordered molecules in the asymmetric unit. Displacement ellipsoids for non-H atoms are drawn at the 20% probability level.

## supplementary crystallographic information

### Comment

Nicotinamide inhibit apoptosis and necrosis by the preservation of membrane phosphatidyl- serine asymmetry (Gaudineau and Auclair, 2004). Nicotinamide derivatives are important pharmaceutical compounds.They exhibit diverse bioactivities such as antimycobacterial (Jaju *et al.*, 2009),antimicrobial (Patel and Shaikh, 2010), CB_2_ receptor agonist (Mitchell *et al.*, 2009) properties. On the other hand 4-thiazolidinone derivatives have been shown to exhibit antifungal (Karali *et al.*, 1998; Ozkirimli *et al.*, 2009) reverse transcriptase inhibitor (Barreca *et al*., 2003), hypoglycemic (Joy *et al.*, 2005) antimicrobial (Chen *et al.*, 2009; Güzeldemirci *et al.*, 2010), antiinlammatory (Vigorita *et al.*, 2003) activities. We combine these two moieties as a part of an ongoing project directed towards the design and synthesis of antiviral molecules bearing 4-thiazolidinone and pyridine-3-carboxamide scaffolds together.

The title compound (I), (Fig. 1), crystallizes in space group P-1 with two crystallographically independent molecules per asymmetric unit. The geometries of the two molecules of (I) are very similar.

In the 1,3-thiazolidine rings of both the A and B molecules, the carboxyl oxygens, the sulfur atoms, the methyl groups and the ring carbon attached to the methyl groups are disordered with site occupancies of 0.509 (7) and 0.491 (7) for S1, molecule A, and site occupancies of 0.464 (14) and 0.536 (14) for S2, molecule B.

In the 1,3-thiazolidine groups of (I), the dihedral angles between the mean planes of two components of the disordered rings are 10.8 (5)° for molecule A and 15.2 (5)° for molecule B.

The benzene and pyridine rings make dihedral angles of 83.9 (2) and 84.88 (19)° for molecules A and B, respectively.

Intramolecular C—H···N hydrogen bonding influences the molecular conformations. The crystal structure is stabilized by the intermolecular N—H···N, C—H···O hydrogen bonds and C—H···Cl interactions. In addition, there is a π-π stacking interaction between the pyridine and benzene rings [*Cg*4···*Cg*7(1 - *x*, 1 - *y*, -*z*) = 3.794 (3) Å, *Cg*4 and *Cg*7 are the centroids of the C1–C6 benzene and N6/C28–C32 pyridine rings].

### Experimental

0.01 mol of *N*'-(4-chlorobenzylidine)pyridine-3-carbohydrazide was reacted with 0.028 mol of 2-mercaptopropanoic acid in anhydrous benzene for 10 h using a Dean-Stark trap. Excess benzene was removed under reduced pressure. The residue was triturated with saturated sodium bicarbonate solution. The separated solid was filtered, washed with water and crystallized from methanol. White crystalline *N*-[5-methyl-2-(4-chlorophenyl)-4-oxo-1,3-thiazolidin-3-yl]pyridine-3-carboxamide. Yield: 56.48%; m.p.: 443.1–447.4 K. UV (EtOH) λ max: 202.8, 220.6, 257.2 nm. IR (KBr) υ: 1673 (amide C=O), 1728 (thia C=O) cm^-1^; ^1^H-NMR (DMSO-d_6_, 500 MHz): 1.55 (3*H*, d, J=7.0 Hz, CH_3_-thia.), 4.13 (1*H*, q, J= 7.0 Hz, H5-thia.), 4.23 (1*H*, dq, J=6.8, 1.47 Hz, H5-thia.), 5.92 (1*H*, s, H2-thia), 7.43–7.47 (2*H*, m, 2-C_6_H_4_-(H2,6)-thia.), 7.48–7.50 (1*H*, m, H5-pyridine), 7.52–7.53 (2*H*, m, 2-C_6_H_4_-(H3,5)-thia.), 8.04–8.09 (1*H*, m, H4-pyridine), 8.73 (1*H*, dd, J_=_6.3 Hz, 1.4 Hz, H6-pyridine), 8.84, 8.85 (1*H*, 2 d, J=2.9, 1.4 Hz, H2-pyridine), 10.94 (1*H*, s, CONH) p.p.m.; ESI– (m/*z*, relative abundance): 347.68 ([M—H+2]^-^, 25.16), 345.56 ([M—H]^-^, 100). Analysis calculated for C_16_H_14_ClN_3_O_2_S. 0.5 EtOH: C 55.06, H 4.62, N 11.48%. Found: C 55.19, H 5.15, N 12.02%.

### Refinement

The NH H atoms were found from a difference Fourier map and restrained to 0.86 (2) Å, and refined with *U*_iso_(H) = 1.2*U*_eq_(N). The C-bound H atoms were geometrically placed (C—H = 0.93–0.98 Å) and refined as riding with *U*_iso_(H) = 1.2 or 1.5*U*_eq_(C). In the 1,3-thiazolidine groups of the two molecules of (I) in the asymmetric unit, the carboxyl O atoms, the sulfur atoms and the methyl groups and the C atoms of the mentioned ring attached to the methyl groups are disoder with site occupancies of 0.509 (7) and 0.491 (7) for the molecule with S1, and with site occupancies of 0.464 (14) and 0.536 (14) for the molecule with S2.

### Crystal data

**Table:**

C_16_H_14_ClN_3_O_2_S	*Z* = 4
*M**_r_* = 347.82	*F*(000) = 720
Triclinic, *P*1	*D*_x_ = 1.374 Mg m^−^^3^
Hall symbol: -P 1	Mo *K*α radiation, λ = 0.71073 Å
*a* = 11.4481 (7) Å	Cell parameters from 29172 reflections
*b* = 12.0276 (7) Å	θ = 1.8–28.1°
*c* = 12.5556 (7) Å	µ = 0.36 mm^−^^1^
α = 91.798 (5)°	*T* = 296 K
β = 100.282 (5)°	Block, colourless
γ = 98.068 (5)°	0.75 × 0.46 × 0.24 mm
*V* = 1681.39 (17) Å^3^	

### Data collection

**Table:**

Stoe IPDS 2 diffractometer	7719 independent reflections
Radiation source: sealed X-ray tube, 12 x 0.4 mm long-fine focus	3832 reflections with *I* > 2σ(*I*)
plane graphite	*R*_int_ = 0.079
Detector resolution: 6.67 pixels mm^-1^	θ_max_ = 27.7°, θ_min_ = 2.2°
ω scans	*h* = −14→14
Absorption correction: integration (*X-RED32*; Stoe & Cie, 2002)	*k* = −15→15
*T*_min_ = 0.772, *T*_max_ = 0.918	*l* = −16→16
27641 measured reflections	

### Refinement

**Table:**

Refinement on *F*^2^	Primary atom site location: structure-invariant direct methods
Least-squares matrix: full	Secondary atom site location: difference Fourier map
*R*[*F*^2^ > 2σ(*F*^2^)] = 0.079	Hydrogen site location: inferred from neighbouring sites
*wR*(*F*^2^) = 0.225	H atoms treated by a mixture of independent and constrained refinement
*S* = 1.04	*w* = 1/[σ^2^(*F*_o_^2^) + (0.1067*P*)^2^] where *P* = (*F*_o_^2^ + 2*F*_c_^2^)/3
7719 reflections	(Δ/σ)_max_ < 0.001
472 parameters	Δρ_max_ = 0.66 e Å^−^^3^
22 restraints	Δρ_min_ = −0.35 e Å^−^^3^

### Special details

**Table:**

Geometry. Bond distances, angles *etc*. have been calculated using the rounded fractional coordinates. All su's are estimated from the variances of the (full) variance-covariance matrix. The cell e.s.d.'s are taken into account in the estimation of distances, angles and torsion angles
Refinement. Refinement on *F*^2^ for ALL reflections except those flagged by the user for potential systematic errors. Weighted *R*-factors *wR* and all goodnesses of fit *S* are based on *F*^2^, conventional *R*-factors *R* are based on *F*, with *F* set to zero for negative *F*^2^. The observed criterion of *F*^2^ > σ(*F*^2^) is used only for calculating -*R*-factor-obs *etc*. and is not relevant to the choice of reflections for refinement. *R*-factors based on *F*^2^ are statistically about twice as large as those based on *F*, and *R*-factors based on ALL data will be even larger.

### Fractional atomic coordinates and isotropic or equivalent isotropic displacement parameters (Å^2^)

**Table:**

	*x*	*y*	*z*	*U*_iso_*/*U*_eq_	Occ. (<1)
Cl1	0.8901 (3)	0.6468 (2)	−0.35513 (17)	0.1812 (13)	
S1A	0.7796 (8)	0.5821 (6)	0.1573 (6)	0.138 (3)	0.509 (7)
O1A	0.6878 (18)	0.2824 (10)	0.2212 (17)	0.144 (7)	0.509 (7)
O2	0.4901 (3)	0.2917 (3)	−0.0056 (4)	0.1164 (16)	
N1	0.7159 (3)	0.3872 (2)	0.0735 (3)	0.0690 (11)	
N2	0.6874 (3)	0.2946 (2)	0.0007 (3)	0.0648 (11)	
N3	0.6148 (3)	0.0002 (3)	−0.2017 (3)	0.0734 (12)	
C1	0.8487 (4)	0.4981 (4)	−0.0856 (4)	0.0818 (17)	
C2	0.8909 (5)	0.5318 (5)	−0.1736 (5)	0.103 (2)	
C3	0.8331 (6)	0.5976 (5)	−0.2426 (4)	0.099 (2)	
C4	0.7303 (6)	0.6322 (4)	−0.2259 (4)	0.099 (2)	
C5	0.6829 (4)	0.5983 (4)	−0.1331 (4)	0.0840 (17)	
C6	0.7462 (4)	0.5298 (3)	−0.0607 (3)	0.0644 (11)	
C7	0.6970 (4)	0.4995 (3)	0.0387 (3)	0.0752 (16)	
C8A	0.7651 (11)	0.4770 (7)	0.2554 (8)	0.1041 (16)	0.509 (7)
C9	0.7211 (6)	0.3722 (4)	0.1807 (4)	0.1041 (16)	
C10A	0.6693 (11)	0.4777 (8)	0.3215 (8)	0.1041 (16)	0.509 (7)
C11	0.5725 (4)	0.2482 (3)	−0.0301 (3)	0.0680 (14)	
C12	0.5490 (3)	0.1396 (3)	−0.0971 (3)	0.0580 (12)	
C13	0.4367 (4)	0.0760 (4)	−0.1111 (4)	0.0764 (16)	
C14	0.4137 (4)	−0.0244 (4)	−0.1702 (4)	0.0826 (17)	
C15	0.5063 (4)	−0.0598 (3)	−0.2123 (3)	0.0754 (16)	
C16	0.6341 (3)	0.0984 (3)	−0.1448 (3)	0.0619 (12)	
O1B	0.7422 (14)	0.2805 (11)	0.2169 (16)	0.097 (5)	0.491 (7)
C8B	0.7113 (12)	0.4845 (6)	0.2354 (7)	0.1041 (16)	0.491 (7)
S1B	0.7748 (4)	0.5927 (4)	0.1577 (4)	0.0654 (15)	0.491 (7)
C10B	0.7736 (12)	0.4965 (8)	0.3510 (7)	0.1041 (16)	0.491 (7)
Cl2	0.58109 (15)	0.34663 (14)	0.67393 (16)	0.1325 (7)	
S2B	0.2083 (8)	−0.1467 (6)	0.5623 (5)	0.0859 (16)	0.536 (14)
O3B	0.1523 (12)	−0.2164 (15)	0.2601 (9)	0.077 (3)	0.536 (14)
O4	−0.0346 (3)	−0.0194 (3)	0.2833 (3)	0.0987 (15)	
N4	0.1814 (3)	−0.0645 (3)	0.3760 (3)	0.0704 (11)	
N5	0.1663 (3)	0.0090 (3)	0.2930 (3)	0.0678 (12)	
N6	−0.0872 (3)	0.2001 (3)	0.0369 (3)	0.0813 (14)	
C17	0.2708 (4)	0.1416 (3)	0.6150 (3)	0.0731 (16)	
C18	0.3620 (4)	0.2248 (4)	0.6595 (4)	0.0828 (17)	
C19	0.4657 (4)	0.2409 (4)	0.6175 (4)	0.0824 (16)	
C20	0.4777 (4)	0.1736 (4)	0.5301 (4)	0.0919 (17)	
C21	0.3865 (4)	0.0890 (4)	0.4873 (4)	0.0815 (17)	
C22	0.2809 (4)	0.0725 (3)	0.5280 (3)	0.0665 (12)	
C23	0.1813 (4)	−0.0218 (3)	0.4866 (3)	0.0732 (16)	
C24B	0.1630 (9)	−0.2483 (8)	0.4489 (6)	0.073 (4)	0.536 (14)
C25	0.1511 (5)	−0.1769 (4)	0.3519 (3)	0.0892 (19)	
C26B	0.0360 (10)	−0.3105 (13)	0.4355 (10)	0.109 (5)	0.536 (14)
C27	0.0520 (4)	0.0210 (3)	0.2470 (3)	0.0698 (14)	
C28	0.0399 (3)	0.0915 (3)	0.1506 (3)	0.0609 (12)	
C29	0.1235 (4)	0.1093 (3)	0.0831 (3)	0.0686 (14)	
C30	0.1012 (4)	0.1714 (3)	−0.0052 (4)	0.0742 (16)	
C31	−0.0038 (4)	0.2152 (3)	−0.0245 (4)	0.0777 (16)	
C32	−0.0642 (4)	0.1396 (4)	0.1230 (4)	0.0744 (16)	
O3A	0.1055 (17)	−0.222 (2)	0.2615 (11)	0.112 (6)	0.464 (14)
S2A	0.1797 (13)	−0.1417 (6)	0.5686 (7)	0.124 (4)	0.464 (14)
C26A	0.125 (2)	−0.3496 (9)	0.4571 (12)	0.129 (8)	0.464 (14)
C24A	0.1067 (14)	−0.2284 (8)	0.4507 (8)	0.084 (5)	0.464 (14)
H4	0.69110	0.67770	−0.27450	0.1190*	
H5	0.61180	0.62040	−0.12010	0.1000*	
H7	0.61160	0.50710	0.02850	0.0900*	
H8A	0.84280	0.47200	0.30120	0.1250*	0.509 (7)
H10A	0.68990	0.54090	0.37330	0.1560*	0.509 (7)
H10B	0.66180	0.40940	0.35890	0.1560*	0.509 (7)
H2	0.96150	0.50930	−0.18740	0.1230*	
H13	0.37660	0.10150	−0.08030	0.0920*	
H14	0.33810	−0.06750	−0.18170	0.1000*	
H15	0.49220	−0.12940	−0.25030	0.0900*	
H16	0.70960	0.14150	−0.13690	0.0740*	
H1	0.89030	0.45170	−0.03990	0.0980*	
HN2	0.749 (2)	0.262 (3)	0.002 (3)	0.0740*	
H10C	0.59440	0.48320	0.27480	0.1560*	0.509 (7)
H8B	0.62630	0.49040	0.23240	0.1250*	0.491 (7)
H10D	0.76410	0.56750	0.38270	0.1560*	0.491 (7)
H10E	0.85750	0.49290	0.35460	0.1560*	0.491 (7)
H10F	0.73940	0.43670	0.39000	0.1560*	0.491 (7)
H20	0.54700	0.18550	0.50050	0.1100*	
H21	0.39560	0.04200	0.43000	0.0980*	
H18	0.35390	0.27040	0.71810	0.1000*	
H24B	0.22180	−0.29980	0.44640	0.0880*	0.536 (14)
H26D	0.03210	−0.36400	0.49030	0.1630*	0.536 (14)
H23	0.10540	0.00730	0.48570	0.0880*	
H26F	−0.01820	−0.25770	0.44240	0.1630*	0.536 (14)
H29	0.19460	0.07880	0.09810	0.0820*	
H30	0.15640	0.18380	−0.05120	0.0890*	
H31	−0.01770	0.25830	−0.08440	0.0930*	
H32	−0.12120	0.12880	0.16750	0.0890*	
HN5	0.226 (3)	0.029 (4)	0.261 (4)	0.0970*	
H26E	0.01380	−0.34920	0.36510	0.1630*	0.536 (14)
H17	0.20090	0.13130	0.64370	0.0870*	
H24A	0.02040	−0.22560	0.44160	0.1010*	0.464 (14)
H26A	0.09860	−0.37910	0.52040	0.1940*	0.464 (14)
H26B	0.20830	−0.35500	0.46130	0.1940*	0.464 (14)
H26C	0.07890	−0.39210	0.39360	0.1940*	0.464 (14)

### Atomic displacement parameters (Å^2^)

**Table:**

	*U*^11^	*U*^22^	*U*^33^	*U*^12^	*U*^13^	*U*^23^
Cl1	0.269 (3)	0.1774 (18)	0.1155 (14)	0.0062 (18)	0.1042 (17)	0.0163 (12)
S1A	0.244 (8)	0.073 (4)	0.091 (5)	−0.012 (4)	0.048 (5)	−0.018 (3)
O1A	0.24 (2)	0.084 (7)	0.102 (7)	−0.052 (8)	0.075 (13)	−0.005 (5)
O2	0.076 (2)	0.101 (2)	0.178 (4)	0.0148 (18)	0.047 (2)	−0.045 (2)
N1	0.087 (2)	0.0497 (16)	0.070 (2)	0.0071 (15)	0.0170 (17)	−0.0032 (14)
N2	0.059 (2)	0.0561 (17)	0.079 (2)	0.0097 (15)	0.0137 (17)	−0.0104 (15)
N3	0.068 (2)	0.077 (2)	0.074 (2)	0.0073 (17)	0.0164 (17)	−0.0166 (17)
C1	0.072 (3)	0.084 (3)	0.093 (3)	0.018 (2)	0.020 (2)	0.003 (2)
C2	0.097 (4)	0.105 (4)	0.115 (4)	0.013 (3)	0.048 (4)	−0.008 (3)
C3	0.119 (4)	0.100 (4)	0.080 (3)	0.001 (3)	0.041 (3)	−0.013 (3)
C4	0.130 (5)	0.084 (3)	0.075 (3)	0.009 (3)	0.003 (3)	0.011 (2)
C5	0.077 (3)	0.076 (3)	0.101 (3)	0.016 (2)	0.022 (3)	−0.015 (2)
C6	0.068 (2)	0.0543 (19)	0.069 (2)	0.0017 (18)	0.016 (2)	−0.0098 (17)
C7	0.075 (3)	0.067 (2)	0.088 (3)	0.013 (2)	0.026 (2)	−0.002 (2)
C8A	0.156 (4)	0.0762 (19)	0.077 (2)	−0.011 (2)	0.037 (3)	−0.0049 (17)
C9	0.156 (4)	0.0762 (19)	0.077 (2)	−0.011 (2)	0.037 (3)	−0.0049 (17)
C10A	0.156 (4)	0.0762 (19)	0.077 (2)	−0.011 (2)	0.037 (3)	−0.0049 (17)
C11	0.062 (2)	0.064 (2)	0.083 (3)	0.0152 (19)	0.024 (2)	−0.0036 (19)
C12	0.050 (2)	0.060 (2)	0.064 (2)	0.0084 (16)	0.0107 (17)	0.0035 (16)
C13	0.057 (2)	0.085 (3)	0.089 (3)	0.005 (2)	0.024 (2)	−0.004 (2)
C14	0.066 (3)	0.085 (3)	0.090 (3)	−0.012 (2)	0.017 (2)	−0.009 (2)
C15	0.081 (3)	0.066 (2)	0.073 (3)	−0.006 (2)	0.013 (2)	−0.0091 (19)
C16	0.052 (2)	0.065 (2)	0.066 (2)	0.0027 (17)	0.0100 (17)	−0.0077 (17)
O1B	0.137 (10)	0.072 (6)	0.085 (7)	0.009 (5)	0.032 (6)	0.026 (4)
C8B	0.156 (4)	0.0762 (19)	0.077 (2)	−0.011 (2)	0.037 (3)	−0.0049 (17)
S1B	0.091 (3)	0.0448 (18)	0.065 (3)	0.0036 (16)	0.034 (2)	−0.0074 (16)
C10B	0.156 (4)	0.0762 (19)	0.077 (2)	−0.011 (2)	0.037 (3)	−0.0049 (17)
Cl2	0.1166 (12)	0.1148 (11)	0.1481 (14)	−0.0239 (9)	0.0171 (10)	−0.0426 (10)
S2B	0.106 (3)	0.097 (3)	0.053 (2)	0.0045 (19)	0.015 (2)	0.0232 (19)
O3B	0.082 (7)	0.073 (5)	0.066 (5)	−0.007 (5)	0.001 (4)	−0.008 (3)
O4	0.0670 (18)	0.121 (3)	0.112 (3)	−0.0004 (17)	0.0334 (18)	0.035 (2)
N4	0.081 (2)	0.071 (2)	0.0566 (18)	0.0041 (17)	0.0111 (16)	0.0054 (15)
N5	0.064 (2)	0.077 (2)	0.063 (2)	0.0064 (17)	0.0149 (16)	0.0156 (16)
N6	0.067 (2)	0.081 (2)	0.092 (3)	0.0213 (18)	−0.003 (2)	−0.001 (2)
C17	0.078 (3)	0.075 (2)	0.074 (3)	0.016 (2)	0.032 (2)	−0.001 (2)
C18	0.099 (3)	0.072 (3)	0.083 (3)	0.016 (2)	0.032 (3)	−0.012 (2)
C19	0.086 (3)	0.070 (2)	0.086 (3)	0.002 (2)	0.012 (2)	−0.012 (2)
C20	0.076 (3)	0.102 (3)	0.096 (3)	−0.001 (3)	0.028 (3)	−0.024 (3)
C21	0.073 (3)	0.094 (3)	0.078 (3)	0.008 (2)	0.023 (2)	−0.021 (2)
C22	0.071 (2)	0.071 (2)	0.061 (2)	0.0129 (19)	0.0199 (19)	0.0038 (18)
C23	0.081 (3)	0.078 (3)	0.061 (2)	0.002 (2)	0.022 (2)	0.0031 (19)
C24B	0.067 (6)	0.077 (7)	0.073 (6)	0.017 (5)	0.002 (5)	0.018 (4)
C25	0.113 (4)	0.075 (3)	0.066 (3)	−0.005 (3)	−0.005 (3)	0.002 (2)
C26B	0.107 (9)	0.105 (10)	0.108 (8)	−0.011 (7)	0.018 (7)	0.036 (7)
C27	0.056 (2)	0.077 (2)	0.077 (3)	0.0028 (19)	0.019 (2)	0.005 (2)
C28	0.051 (2)	0.059 (2)	0.070 (2)	0.0035 (16)	0.0079 (18)	0.0024 (17)
C29	0.064 (2)	0.071 (2)	0.075 (3)	0.0182 (19)	0.016 (2)	0.015 (2)
C30	0.080 (3)	0.072 (2)	0.073 (3)	0.015 (2)	0.017 (2)	0.009 (2)
C31	0.090 (3)	0.063 (2)	0.073 (3)	0.013 (2)	−0.005 (2)	0.0019 (19)
C32	0.057 (2)	0.080 (3)	0.086 (3)	0.008 (2)	0.016 (2)	−0.003 (2)
O3A	0.131 (15)	0.106 (8)	0.077 (7)	−0.020 (11)	−0.007 (7)	−0.005 (5)
S2A	0.207 (10)	0.088 (3)	0.067 (3)	−0.042 (4)	0.049 (4)	−0.006 (2)
C26A	0.22 (2)	0.069 (8)	0.104 (10)	0.010 (9)	0.049 (12)	0.019 (6)
C24A	0.088 (11)	0.066 (7)	0.095 (8)	0.012 (8)	0.005 (7)	0.011 (5)

### Geometric parameters (Å, °)

**Table:**

Cl1—C3	1.745 (6)	C4—H4	0.9300
Cl2—C19	1.733 (5)	C5—H5	0.9300
S1A—C7	1.800 (8)	C7—H7	0.9800
S1A—C8A	1.804 (12)	C8A—H8A	0.9800
S1B—C8B	1.800 (10)	C8B—H8B	0.9800
S1B—C7	1.850 (6)	C10A—H10B	0.9600
S2A—C24A	1.787 (13)	C10A—H10A	0.9600
S2A—C23	1.798 (8)	C10A—H10C	0.9600
S2B—C24B	1.800 (11)	C10B—H10E	0.9600
S2B—C23	1.836 (8)	C10B—H10F	0.9600
O1A—C9	1.248 (15)	C10B—H10D	0.9600
O1B—C9	1.244 (15)	C13—H13	0.9300
O2—C11	1.221 (6)	C14—H14	0.9300
O3A—C25	1.235 (16)	C15—H15	0.9300
O3B—C25	1.236 (13)	C16—H16	0.9300
O4—C27	1.211 (6)	C17—C22	1.383 (5)
N1—C7	1.465 (4)	C17—C18	1.366 (6)
N1—C9	1.356 (6)	C18—C19	1.375 (7)
N1—N2	1.382 (4)	C19—C20	1.381 (7)
N2—C11	1.338 (6)	C20—C21	1.372 (7)
N3—C15	1.328 (6)	C21—C22	1.386 (7)
N3—C16	1.331 (5)	C22—C23	1.498 (6)
N2—HN2	0.85 (3)	C24A—C25	1.538 (12)
N4—C23	1.466 (5)	C24A—C26A	1.504 (16)
N4—C25	1.358 (6)	C24B—C25	1.510 (9)
N4—N5	1.391 (5)	C24B—C26B	1.517 (16)
N5—C27	1.362 (6)	C27—C28	1.497 (5)
N6—C31	1.327 (6)	C28—C29	1.387 (6)
N6—C32	1.330 (6)	C28—C32	1.391 (6)
N5—HN5	0.86 (4)	C29—C30	1.363 (6)
C1—C2	1.335 (8)	C30—C31	1.365 (6)
C1—C6	1.367 (7)	C17—H17	0.9300
C2—C3	1.342 (8)	C18—H18	0.9300
C3—C4	1.350 (10)	C20—H20	0.9300
C4—C5	1.418 (7)	C21—H21	0.9300
C5—C6	1.418 (6)	C23—H23	0.9800
C6—C7	1.493 (6)	C24A—H24A	0.9800
C8A—C9	1.517 (10)	C24B—H24B	0.9800
C8A—C10A	1.490 (17)	C26A—H26A	0.9600
C8B—C10B	1.492 (13)	C26A—H26B	0.9600
C8B—C9	1.523 (10)	C26A—H26C	0.9600
C11—C12	1.496 (5)	C26B—H26D	0.9600
C12—C16	1.371 (5)	C26B—H26E	0.9600
C12—C13	1.380 (6)	C26B—H26F	0.9600
C13—C14	1.365 (7)	C29—H29	0.9300
C14—C15	1.377 (7)	C30—H30	0.9300
C1—H1	0.9300	C31—H31	0.9300
C2—H2	0.9300	C32—H32	0.9300
			
Cl1···C26B^i^	3.373 (13)	C16···O3B^vi^	3.242 (15)
Cl1···H26D^i^	2.7600	C17···O4^iii^	3.367 (6)
Cl1···H26F^i^	3.1100	C18···C13^xi^	3.479 (7)
Cl1···H26A^i^	3.1200	C21···C21^ii^	3.570 (7)
Cl2···H24B^ii^	3.0400	C21···N5	3.195 (6)
S2A···O4^iii^	3.428 (12)	C23···O4	3.225 (5)
S2A···O1A^ii^	3.42 (2)	C25···O4	3.071 (6)
S1A···H18^iv^	3.0600	C26B···Cl1^xii^	3.373 (13)
S1B···H18^iv^	2.9400	C27···O3B	3.222 (18)
S1B···H30^v^	3.1100	C27···O3A	3.07 (2)
S2B···H20^ii^	3.1300	C29···C4^v^	3.578 (6)
O1A···N2	2.78 (2)	C31···O3B^viii^	3.139 (13)
O1A···C11	3.19 (2)	C31···O3A^viii^	3.004 (15)
O1A···C14^vi^	3.160 (14)	C32···O1B^ix^	3.322 (17)
O1A···S2A^ii^	3.42 (2)	C1···H1^xiii^	3.0900
O1A···C15^vi^	3.216 (16)	C16···HN2	2.68 (4)
O1B···N2	2.69 (2)	C16···H29^vi^	3.0900
O1B···C11	3.33 (2)	C16···HN5^vi^	2.90 (4)
O1B···C32^vii^	3.322 (17)	C18···H10D^iv^	3.0700
O1B···C14^vi^	3.315 (15)	C18···H10A^iv^	2.9900
O2···N1	2.677 (5)	C21···HN5	3.10 (5)
O2···C7	3.154 (5)	C27···H23	2.9600
O2···C9	3.215 (7)	C29···HN5	2.61 (5)
O2···C5^v^	3.245 (6)	C31···HN2^ix^	3.03 (3)
O3A···C31^viii^	3.004 (15)	C32···HN2^ix^	2.99 (3)
O3A···O4	3.13 (2)	H1···N2	2.9100
O3A···C27	3.07 (2)	H1···C1^xiii^	3.0900
O3A···N5	2.77 (2)	H1···N1	2.7000
O3B···C27	3.222 (18)	HN2···O1B	2.72 (4)
O3B···C31^viii^	3.139 (13)	HN2···N6^vii^	2.09 (3)
O3B···N5	2.707 (18)	HN2···C31^vii^	3.03 (3)
O3B···C16^vi^	3.242 (15)	HN2···C16	2.68 (4)
O4···O3A	3.13 (2)	HN2···C32^vii^	2.99 (3)
O4···S2A^iii^	3.428 (12)	HN2···H16	2.1700
O4···C25	3.071 (6)	HN5···H29	2.1300
O4···C23	3.225 (5)	HN5···C29	2.61 (5)
O4···N4	2.674 (5)	HN5···H21	2.5900
O4···C17^iii^	3.367 (6)	HN5···N3^vi^	2.16 (4)
O1A···H10C	2.8800	HN5···C21	3.10 (5)
O1A···H15^vi^	2.6500	HN5···C16^vi^	2.90 (4)
O1A···H14^vi^	2.5800	H5···H7	2.3400
O1A···H10B	2.3600	H5···O2^v^	2.4200
O1B···H32^vii^	2.6900	H7···H7^v^	2.5100
O1B···H14^vi^	2.5900	H7···H8B	2.5500
O1B···H10F	2.8400	H7···H5	2.3400
O1B···HN2	2.72 (4)	H7···O2^v^	2.8300
O2···H7^v^	2.8300	H7···O2	2.7500
O2···H5^v^	2.4200	H8A···H26A^ii^	2.5600
O2···H13	2.5300	H8B···H7	2.5500
O2···H7	2.7500	H10A···C18^iv^	2.9900
O3A···H26C	2.6900	H10B···O1A	2.3600
O3A···H31^viii^	2.2700	H10C···O1A	2.8800
O3B···H31^viii^	2.4500	H10D···C18^iv^	3.0700
O3B···H26E	2.6400	H10F···O1B	2.8400
O3B···H16^vi^	2.5000	H13···O2	2.5300
O4···H17^iii^	2.5000	H14···H32^viii^	2.5300
O4···H32	2.5300	H14···O1B^vi^	2.5900
O4···H23	2.7300	H14···O1A^vi^	2.5800
N1···O2	2.677 (5)	H15···O1A^vi^	2.6500
N2···C1	3.192 (6)	H16···N2	2.5600
N2···O1A	2.78 (2)	H16···N6^vii^	2.8800
N2···N6^vii^	2.932 (5)	H16···HN2	2.1700
N2···O1B	2.69 (2)	H16···O3B^vi^	2.5000
N3···N5^vi^	2.950 (5)	H17···H23	2.4400
N4···O4	2.674 (5)	H17···O4^iii^	2.5000
N5···N3^vi^	2.950 (5)	H18···S1A^iv^	3.0600
N5···C21	3.195 (6)	H18···S1B^iv^	2.9400
N5···O3A	2.77 (2)	H20···S2B^ii^	3.1300
N5···O3B	2.707 (18)	H21···HN5	2.5900
N6···N2^ix^	2.932 (5)	H21···N4	2.5700
N1···H1	2.7000	H21···N5	2.8400
N2···H1	2.9100	H21···N3^vi^	2.8700
N2···H16	2.5600	H23···H17	2.4400
N3···H29^vi^	2.6500	H23···O4	2.7300
N3···HN5^vi^	2.16 (4)	H23···C27	2.9600
N3···H21^vi^	2.8700	H23···H23^iii^	2.4900
N4···H21	2.5700	H24B···Cl2^ii^	3.0400
N5···H21	2.8400	H26A···H8A^ii^	2.5600
N5···H29	2.6700	H26A···Cl1^xii^	3.1200
N6···HN2^ix^	2.09 (3)	H26C···O3A	2.6900
N6···H16^ix^	2.8800	H26D···Cl1^xii^	2.7600
C1···N2	3.192 (6)	H26E···O3B	2.6400
C4···C29^v^	3.578 (6)	H26F···Cl1^xii^	3.1100
C5···O2^v^	3.245 (6)	H29···N5	2.6700
C7···O2	3.154 (5)	H29···HN5	2.1300
C9···O2	3.215 (7)	H29···C16^vi^	3.0900
C11···O1A	3.19 (2)	H29···N3^vi^	2.6500
C11···O1B	3.33 (2)	H30···S1B^v^	3.1100
C13···C13^vi^	3.584 (7)	H31···O3A^viii^	2.2700
C13···C18^x^	3.479 (7)	H31···O3B^viii^	2.4500
C14···O1A^vi^	3.160 (14)	H32···H14^viii^	2.5300
C14···O1B^vi^	3.315 (15)	H32···O4	2.5300
C15···O1A^vi^	3.216 (16)	H32···O1B^ix^	2.6900
			
C7—S1A—C8A	98.3 (5)	C8B—C10B—H10D	109.00
C7—S1B—C8B	84.7 (4)	C12—C13—H13	120.00
C23—S2A—C24A	88.9 (5)	C14—C13—H13	120.00
C23—S2B—C24B	97.0 (4)	C15—C14—H14	121.00
N2—N1—C7	121.0 (3)	C13—C14—H14	121.00
C7—N1—C9	115.8 (3)	C14—C15—H15	118.00
N2—N1—C9	119.3 (3)	N3—C15—H15	118.00
N1—N2—C11	119.4 (3)	C12—C16—H16	118.00
C15—N3—C16	117.2 (3)	N3—C16—H16	118.00
C11—N2—HN2	127 (2)	C18—C17—C22	121.1 (4)
N1—N2—HN2	109 (2)	C17—C18—C19	119.9 (4)
N5—N4—C23	118.1 (3)	Cl2—C19—C18	119.8 (4)
N5—N4—C25	119.1 (3)	C18—C19—C20	120.2 (4)
C23—N4—C25	118.2 (3)	Cl2—C19—C20	120.0 (4)
N4—N5—C27	117.7 (3)	C19—C20—C21	119.4 (4)
C31—N6—C32	116.8 (4)	C20—C21—C22	121.0 (4)
N4—N5—HN5	118 (3)	C17—C22—C23	118.9 (4)
C27—N5—HN5	122 (3)	C21—C22—C23	122.7 (4)
C2—C1—C6	122.0 (5)	C17—C22—C21	118.4 (4)
C1—C2—C3	121.1 (6)	S2B—C23—C22	109.0 (4)
Cl1—C3—C2	121.6 (5)	S2A—C23—C22	114.3 (5)
C2—C3—C4	121.4 (5)	S2A—C23—N4	106.3 (4)
Cl1—C3—C4	116.9 (4)	S2B—C23—N4	100.7 (3)
C3—C4—C5	119.1 (5)	N4—C23—C22	113.6 (3)
C4—C5—C6	118.5 (5)	S2A—C24A—C26A	113.9 (10)
C5—C6—C7	117.3 (4)	C25—C24A—C26A	111.0 (10)
C1—C6—C7	124.8 (4)	S2A—C24A—C25	108.0 (8)
C1—C6—C5	117.9 (4)	C25—C24B—C26B	100.6 (8)
N1—C7—C6	113.5 (3)	S2B—C24B—C25	103.6 (6)
S1B—C7—C6	111.5 (3)	S2B—C24B—C26B	115.6 (8)
S1B—C7—N1	103.1 (3)	O3B—C25—N4	120.1 (9)
S1A—C7—C6	112.0 (4)	N4—C25—C24B	114.7 (5)
S1A—C7—N1	99.2 (3)	O3A—C25—N4	125.8 (11)
S1A—C8A—C10A	118.9 (8)	O3B—C25—C24B	121.4 (9)
C9—C8A—C10A	102.9 (8)	O3A—C25—C24A	117.6 (11)
S1A—C8A—C9	100.5 (6)	N4—C25—C24A	106.9 (5)
S1B—C8B—C9	106.8 (6)	N5—C27—C28	115.7 (4)
S1B—C8B—C10B	111.3 (7)	O4—C27—C28	121.7 (4)
C9—C8B—C10B	112.1 (8)	O4—C27—N5	122.6 (4)
O1B—C9—C8B	132.6 (10)	C29—C28—C32	116.8 (4)
N1—C9—C8B	107.7 (5)	C27—C28—C29	124.7 (3)
O1A—C9—C8A	118.5 (10)	C27—C28—C32	118.5 (4)
O1B—C9—N1	119.4 (10)	C28—C29—C30	119.9 (4)
O1A—C9—N1	125.8 (10)	C29—C30—C31	118.6 (4)
N1—C9—C8A	115.6 (5)	N6—C31—C30	124.1 (4)
N2—C11—C12	116.7 (4)	N6—C32—C28	124.0 (4)
O2—C11—N2	122.3 (4)	C18—C17—H17	119.00
O2—C11—C12	121.1 (4)	C22—C17—H17	120.00
C13—C12—C16	117.6 (4)	C17—C18—H18	120.00
C11—C12—C13	119.0 (4)	C19—C18—H18	120.00
C11—C12—C16	123.4 (3)	C19—C20—H20	120.00
C12—C13—C14	120.1 (4)	C21—C20—H20	120.00
C13—C14—C15	117.8 (4)	C20—C21—H21	120.00
N3—C15—C14	123.6 (4)	C22—C21—H21	119.00
N3—C16—C12	123.7 (3)	S2B—C23—H23	119.00
C2—C1—H1	119.00	N4—C23—H23	107.00
C6—C1—H1	119.00	C22—C23—H23	107.00
C3—C2—H2	119.00	S2A—C23—H23	107.00
C1—C2—H2	120.00	S2A—C24A—H24A	108.00
C3—C4—H4	121.00	C26A—C24A—H24A	108.00
C5—C4—H4	120.00	C25—C24A—H24A	108.00
C4—C5—H5	121.00	S2B—C24B—H24B	112.00
C6—C5—H5	121.00	C25—C24B—H24B	112.00
S1A—C7—H7	111.00	C26B—C24B—H24B	112.00
S1B—C7—H7	107.00	C24A—C26A—H26A	109.00
N1—C7—H7	111.00	C24A—C26A—H26B	110.00
C6—C7—H7	111.00	H26A—C26A—H26B	110.00
S1A—C8A—H8A	111.00	H26A—C26A—H26C	109.00
C10A—C8A—H8A	111.00	C24A—C26A—H26C	109.00
C9—C8A—H8A	111.00	H26B—C26A—H26C	110.00
C9—C8B—H8B	109.00	C24B—C26B—H26E	109.00
C10B—C8B—H8B	109.00	C24B—C26B—H26F	109.00
S1B—C8B—H8B	109.00	C24B—C26B—H26D	109.00
H10A—C10A—H10B	109.00	H26D—C26B—H26F	110.00
H10B—C10A—H10C	109.00	H26E—C26B—H26F	109.00
H10A—C10A—H10C	109.00	H26D—C26B—H26E	109.00
C8A—C10A—H10C	109.00	C30—C29—H29	120.00
C8A—C10A—H10B	109.00	C28—C29—H29	120.00
C8A—C10A—H10A	109.00	C31—C30—H30	121.00
C8B—C10B—H10E	109.00	C29—C30—H30	121.00
C8B—C10B—H10F	109.00	N6—C31—H31	118.00
H10D—C10B—H10E	109.00	C30—C31—H31	118.00
H10E—C10B—H10F	109.00	C28—C32—H32	118.00
H10D—C10B—H10F	110.00	N6—C32—H32	118.00
			
C8A—S1A—C7—N1	−26.5 (6)	C1—C6—C7—S1A	76.2 (5)
C8A—S1A—C7—C6	−146.5 (5)	C1—C6—C7—N1	−35.1 (6)
C7—S1A—C8A—C9	13.9 (8)	C5—C6—C7—S1A	−102.5 (5)
C7—S1A—C8A—C10A	−97.4 (8)	C5—C6—C7—N1	146.2 (4)
C23—S2B—C24B—C26B	−98.4 (9)	S1A—C8A—C9—N1	4.6 (9)
C24B—S2B—C23—N4	−19.7 (6)	S1A—C8A—C9—O1A	−172.0 (12)
C24B—S2B—C23—C22	−139.5 (5)	C10A—C8A—C9—N1	127.7 (7)
C23—S2B—C24B—C25	10.6 (7)	C10A—C8A—C9—O1A	−48.9 (14)
C9—N1—N2—C11	79.5 (5)	N2—C11—C12—C16	−13.1 (5)
C9—N1—C7—S1A	34.4 (6)	N2—C11—C12—C13	166.0 (4)
N2—N1—C7—C6	−48.9 (5)	O2—C11—C12—C13	−14.3 (6)
C7—N1—N2—C11	−77.4 (5)	O2—C11—C12—C16	166.6 (4)
C9—N1—C7—C6	153.4 (4)	C13—C12—C16—N3	−1.3 (6)
N2—N1—C9—O1A	−9.6 (14)	C11—C12—C16—N3	177.9 (4)
C7—N1—C9—O1A	148.6 (13)	C11—C12—C13—C14	−178.8 (4)
N2—N1—C9—C8A	174.1 (6)	C16—C12—C13—C14	0.4 (6)
C7—N1—C9—C8A	−27.8 (8)	C12—C13—C14—C15	1.4 (7)
N2—N1—C7—S1A	−167.9 (4)	C13—C14—C15—N3	−2.5 (7)
N1—N2—C11—C12	−172.1 (3)	C18—C17—C22—C23	176.4 (4)
N1—N2—C11—O2	8.2 (6)	C18—C17—C22—C21	0.5 (6)
C15—N3—C16—C12	0.3 (6)	C22—C17—C18—C19	0.2 (7)
C16—N3—C15—C14	1.7 (6)	C17—C18—C19—C20	0.3 (7)
C23—N4—C25—O3B	−178.7 (8)	C17—C18—C19—Cl2	179.9 (4)
N5—N4—C25—C24B	−176.0 (5)	C18—C19—C20—C21	−1.5 (7)
C23—N4—C25—C24B	−20.5 (7)	Cl2—C19—C20—C21	178.9 (4)
N5—N4—C25—O3B	25.9 (10)	C19—C20—C21—C22	2.2 (7)
C23—N4—N5—C27	−85.1 (5)	C20—C21—C22—C23	−177.4 (4)
C25—N4—N5—C27	70.3 (5)	C20—C21—C22—C17	−1.7 (7)
C25—N4—C23—C22	142.4 (4)	C21—C22—C23—N4	−23.6 (6)
N5—N4—C23—C22	−61.9 (5)	C21—C22—C23—S2B	87.8 (5)
C25—N4—C23—S2B	26.0 (5)	C17—C22—C23—N4	160.7 (4)
N5—N4—C23—S2B	−178.3 (4)	C17—C22—C23—S2B	−87.8 (5)
N4—N5—C27—C28	−173.9 (3)	C26B—C24B—C25—N4	122.6 (7)
N4—N5—C27—O4	8.5 (6)	S2B—C24B—C25—N4	2.8 (8)
C32—N6—C31—C30	1.0 (6)	C26B—C24B—C25—O3B	−79.5 (12)
C31—N6—C32—C28	−0.7 (7)	S2B—C24B—C25—O3B	160.6 (9)
C6—C1—C2—C3	−0.9 (9)	N5—C27—C28—C29	24.8 (5)
C2—C1—C6—C7	−177.1 (5)	O4—C27—C28—C29	−157.5 (4)
C2—C1—C6—C5	1.5 (7)	O4—C27—C28—C32	19.8 (6)
C1—C2—C3—C4	0.0 (9)	N5—C27—C28—C32	−157.9 (4)
C1—C2—C3—Cl1	177.5 (5)	C32—C28—C29—C30	0.0 (6)
C2—C3—C4—C5	0.2 (8)	C27—C28—C32—N6	−177.3 (4)
Cl1—C3—C4—C5	−177.5 (4)	C27—C28—C29—C30	177.3 (4)
C3—C4—C5—C6	0.5 (7)	C29—C28—C32—N6	0.2 (7)
C4—C5—C6—C7	177.5 (4)	C28—C29—C30—C31	0.3 (6)
C4—C5—C6—C1	−1.3 (6)	C29—C30—C31—N6	−0.8 (6)

Symmetry codes: (i) *x*+1, *y*+1, *z*−1; (ii) −*x*+1, −*y*, −*z*+1; (iii) −*x*, −*y*, −*z*+1; (iv) −*x*+1, −*y*+1, −*z*+1; (v) −*x*+1, −*y*+1, −*z*; (vi) −*x*+1, −*y*, −*z*; (vii) *x*+1, *y*, *z*; (viii) −*x*, −*y*, −*z*; (ix) *x*−1, *y*, *z*; (x) *x*, *y*, *z*−1; (xi) *x*, *y*, *z*+1; (xii) *x*−1, *y*−1, *z*+1; (xiii) −*x*+2, −*y*+1, −*z*.

### Hydrogen-bond geometry (Å, °)

**Table:**

*D*—H···*A*	*D*—H	H···*A*	*D*···*A*	*D*—H···*A*
N2—HN2···N6^vii^	0.85 (3)	2.09 (3)	2.932 (5)	168 (3)
N5—HN5···N3^vi^	0.86 (4)	2.16 (4)	2.950 (5)	153 (4)
C5—H5···O2^v^	0.93	2.42	3.245 (6)	147
C14—H14···O1A^vi^	0.93	2.58	3.160 (14)	121
C16—H16···N2	0.93	2.56	2.865 (5)	100
C16—H16···O3B^vi^	0.93	2.50	3.242 (15)	136
C17—H17···O4^iii^	0.93	2.50	3.367 (6)	155
C21—H21···N4	0.93	2.57	2.885 (6)	101
C26B—H26D···Cl1^xii^	0.96	2.76	3.373 (13)	123
C31—H31···O3B^viii^	0.93	2.45	3.139 (13)	131

Symmetry codes: (vii) *x*+1, *y*, *z*; (vi) −*x*+1, −*y*, −*z*; (v) −*x*+1, −*y*+1, −*z*; (iii) −*x*, −*y*, −*z*+1; (xii) *x*−1, *y*−1, *z*+1; (viii) −*x*, −*y*, −*z*.
